# Decreased Histone Deacetylase 2 (HDAC2) in Peripheral Blood Monocytes (PBMCs) of COPD Patients

**DOI:** 10.1371/journal.pone.0147380

**Published:** 2016-01-25

**Authors:** Chunting Tan, Lingling Xuan, Shuhua Cao, Ganggang Yu, Qi Hou, Haoyan Wang

**Affiliations:** 1 Department of Respiratory Medicine, Beijing Friendship Hospital, Capital Medical University, Beijing, China; 2 State Key Laboratory of Bioactive Substances and Functions of Natural Medicines, Institute of Materia Medica, Chinese Academy of Medical Sciences and Peking Union Medical College, Beijing, China; 3 State Key Laboratory of Molecular Oncology, Cancer Institute/Hospital, Chinese Academy of Medical Sciences & Peking Union Medical College, Beijing, China; University of Florida, UNITED STATES

## Abstract

**Background:**

Histone deacetylase 2 (HDAC2) is a class I histone deacetylase family member that plays a critical role in suppressing inflammatory gene expression in the airways, lung parenchyma, and alveolar macrophages in patients with chronic obstructive pulmonary disease (COPD). However, the expression of HDAC2 in peripheral blood monocytes (PBMCs), nuclear factor kappa B (NF-κB) p65, and serum inflammatory cytokine levels in COPD patients, smokers, and non-smokers remains unclear.

**Methods:**

PBMCs were obtained from COPD patients, healthy smokers, and healthy nonsmokers. The HDAC2 and NF-κB p65 expression were quantified by Western Blot. HDAC activity was assessed by an HDAC fluorometric immunoprecipitation activity assay kit. Serum tumor necrosis factor-alpha (TNF-α) and interleukin-8 (IL-8) levels were measured by ELISA.

**Results:**

HDAC2 expression and HDAC activity were decreased in PBMCs in COPD patients compared with smokers and non-smokers. Increased NF-κB p65 expression, serum TNF-α and IL-8 levels were observed in COPD patients compared with nonsmokers. The FEV_1_%pred was positively correlated with HDAC2 expression and HDAC activity in COPD patients. Smokers had decreased HDAC activity, increased NF-κB p65 expression and serum TNF-α compared with nonsmokers.

**Conclusions:**

HDAC2 expression was decreased in PBMCs of COPD patients and was correlated with disease severity. The reduction of HDAC2 expression not only directly enhances the expression of inflammatory genes, but may account for the activation of NF-κB mediated inflammation. Decreased HDAC2 may serve as a potential biomarker of COPD and predict the decline of lung function.

## Introduction

Chronic obstructive pulmonary disease (COPD) is characterized by airflow limitation, which is associated with progressive airway inflammation. Some initial studies suggest that the gradual inflammation in the small airways and lung parenchyma is mediated by the expression of multiple biomarkers and inflammatory genes [1–4]. Inflammatory gene expression is regulated by a balance between histone acetylation and deacetylation. In particular, the histone deacetylases (HDACs) play a critical role in suppression of gene expression by reversing the hyperacetylation of core histones [1, 5]. The reduction of HDAC expression and activity can further enhance inflammatory gene expression [6, 7]. So far, a total of 11 classical HDAC isoenzymes has been identified that may be involved in regulating the expression of inflammatory genes and inflammatory signaling pathways. Histone deacetylase 2 (HDAC2), as one of the family members of the class I histone deacetylases, is critical for regulating the expression of inflammatory genes [5, 6, 8]. Previous in vitro studies [6, 8–11] using specimens from COPD patients have demonstrated that HDAC expression and activity were reduced in lung parenchyma, bronchial biopsies, and alveolar macrophages. However, the assessment of HDAC2 expression in peripheral blood monocytes (PBMCs) of COPD patients and smokers has not been conducted.

Additionally, inflammatory genes in the pathogenesis of COPD are also regulated by proinflammatory transcription factors, including nuclear factor kappa B (NF-κB) and activator protein (AP)-1, which could be activated by histone acetylation [12–16]. NF-κB is made up of two subunits (p65, p50) and is a target for acetylation and deacetylation [17–19]. Acetylated p65 can be deacetylated by HDACs and bind to the inhibitor IκB-α within the nucleus, which could terminate the activity of NF-κB [1, 19]. Thus, the reduction of HDAC activity also results in the activation of NF-κB and increases expression of NF-κB mediated proinflammatory genes, including tumor necrosis factor-alpha (TNF-α) and interleukin-8 (IL-8) [12, 20–22]. In this proposed work, we hypothesized that the decreased HDAC2 may cause increased expression of NF-κB and inflammatory cytokines in COPD patients.

Pulmonary function is one of the key indicators for the prognosis and management of COPD. Total HDAC activity in lung tissue is reduced and reflects the severity of COPD [6]. At present, the relationship between HDAC2 and the severity of airway obstruction in COPD patients is unclear. Therefore, we also hypothesized that the decreased HDAC2 is correlated with the severity of COPD.

## Materials and Methods

### Subjects

A total of 30 subjects was divided into three groups. The first group (the cases group) was patients with stable COPD. The second group was current smokers without airway obstruction, and the third group was healthy nonsmokers who had normal lung function and no respiratory symptoms. The presence of COPD was identified by the GOLD criteria guidelines with a post-bronchodilator ratio of forced expiratory volume in 1 second (FEV_1_)/forced vital capacity (FVC) ≤70% [1]. Both COPD patients and smokers had a smoking history of 10 pack-years or more. All subjects had no pulmonary disease, including asthma, tuberculosis, bronchiectasis, lung cancer, and other conditions such as a history of lung surgery and infective or interstitial disease. COPD patients with exacerbation or receiving systemic corticosteroids (oral or intravenous injection therapy) within four weeks prior to the study were excluded. Also, those patients treated with oral theophylline were also excluded [23].

The study was conducted in accordance with the Declaration of Helsinki and approved by the Ethics Committees of Beijing Friendship Hospital. All participants agreed and gave their signed consent prior to the study.

### PBMCs Isolation

Venous blood samples (20ml) were collected in EDTA vacutainer tubes (BD Biosciences). PBMCs were isolated by Ficoll gradient centrifugation according to the instructions of the manufacturer (Ficoll Paque Plus, GE Healthcare, Biosciences AB, Sweden). Cells were washed twice with PBS and suspended in RPMI 1640 supplemented with 10% fetal bovine serum (Hyclone, Logan, UT, USA). Cell viability was assessed by trypan blue method and counted with a hemocytometer.

### Nuclear protein extraction

Nuclear extracts were prepared using the NE-PER Nuclear Extraction Kit according to the manufacturer's directions (Pierce Biotechnology Inc, Rockford, IL, USA). Protein concentration was determined using the BCA Protein Assay Kit (Thermo Scientific, Rockford, IL, USA). The resulting soluble protein and nuclear protein fractions were then stored at -80°C.

### Western Blot

Cell extracts containing 15ug protein were separated on 12% sodium dodecyl sulfate-polyacrylamide gel electrophoresis (SDS-PAGE) and transferred to PVDF membranes (Millipore, Bedford, MA, USA). The membranes were then blocked in Tris-buffered saline (TBS) with 0.1% Tween 20 containing 5% nonfat dry milk for two hours at room temperature. Subsequently, the membranes were incubated with specific primary antibodies for HDAC2 (Cell Signaling Technology, Beverly, MA, USA), NF-κB p65 (Cell Signaling, Beverly, MA, USA) and β-actin (Sigma-Aldrich, St Louis, MO, USA) monoclonal antibodies overnight at 4°C. After washing, the membranes were incubated with secondary antibodies at the appropriate dilutions for 1hour at room temperature and detected using enhanced chemiluminescence ECL substrate kit (Tiangen Biotech, Beijing, China). The density of each band was quantified by QuantiScan Version 11 (Biosoft, Cambridge, UK).

### HDAC activity assay

HDAC activity in the nuclear extract was measured using an HDAC Fluorometric Immunoprecipitation activity assay kit (BioVision Mountainview, CA, USA) according to the manufacturer's instructions. Fluorescence signal was detected with the excitation at 360nm and emission at 460nm using a fluorescence microplate reader (BioTek, Winooski, VT, USA).

### Enzyme-linked immunosorbent assay (ELISA)

Serum levels of TNF-α and IL-8 were measured by ELISA (Biolegend, San Diego, CA, USA) according to the manufacturer’s instructions. The lower detection limit was 2pg/mL for TNF-α and 8pg/mL for IL-8.

### Statistical analysis

Results are expressed as mean ±SD for baseline characteristics of the subjects and mean ±SEM for experimental data. HDAC2 expression, HDAC activity, p65 expression, serum TNF-α and IL-8 level were compared between groups with one-way ANOVA. If the difference was significant, the Tukey-Kramer’s test was performed for multiple comparisons using SPSS 15.0 (SPSS Inc, Chicago, Illinois). Pearson correlations between FEV_1_%pred with HDAC2 expression, HDAC activity, p65 expression, serum TNF-α and IL-8 level were calculated using GraphPad Prism (GraphPad Software Inc, La Jolla, California). A value of P < .05 (two-tailed) was considered statistically significant.

## Results

### Subjects' characteristics

All study subjects were recruited for the study between March 2013 and August 2013. The demographic data from all participants are presented in Table 1. All COPD patients were habitual cigarette smokers, of whom 2 were ex-smokers while the others were current smokers. COPD patients included 5 in GOLD stage 2, 4 in stage 3, and 1 in stage 4. Firstly, the comparisons of clinical features among COPD patients, healthy smokers, and nonsmokers were performed. No significant differences were observed among COPD patients, smokers, and nonsmokers regarding age, height, and body weight. Significantly higher pack-years of cigarette smoking was observed in COPD compared with smokers (38.6±18.8 vs. 21.3±10.0, P<0.01) and nonsmokers (38.6±18.8 vs. 0, P<0.001).

**Table 1:** Baseline characteristics of the study subjects.*

	COPD (n = 10)	Smokers (n = 10)	Nonsmokers (n = 10)
Age, y	65±3	50±5	59±7
Sex, M/F	6/4	7/3	4/6
Height, cm	166.4±2.1	168.3±3.8	164.4±3.4
Weight, kg	66.5±2.7	70.9±4.1	65.2±3.7
Smoking(pack-years)	38.6±18.8	21.3±10.0	0
FEV_1_, L	1.4±0.1	2.8±0.3	2.5±0.2
FEV_1_%pred	51.9±12.6	101.4±8.4	93.6±4.7
FVC, L	2.9±0.2	3.5±0.4	3.0±0.2
FVC%pred	81.8±4.8	100.3±6.9	93.4±4.8
FEV_1_/FVC, %	47.8±11.2	81.4±2.1	84.0±0.9

* COPD: chronic obstructive pulmonary disease, FEV_1_: forced expiratory volume in one second, FVC: forced vital capacity, pack-years: the number of packed cigarettes smoked per day multiplied by the number of years of smoking (one pack = 20 cigarettes). Data are presented as the mean ±SD.

### Decreased HDAC2 expression and HDAC activity in COPD

**Fig 1:**
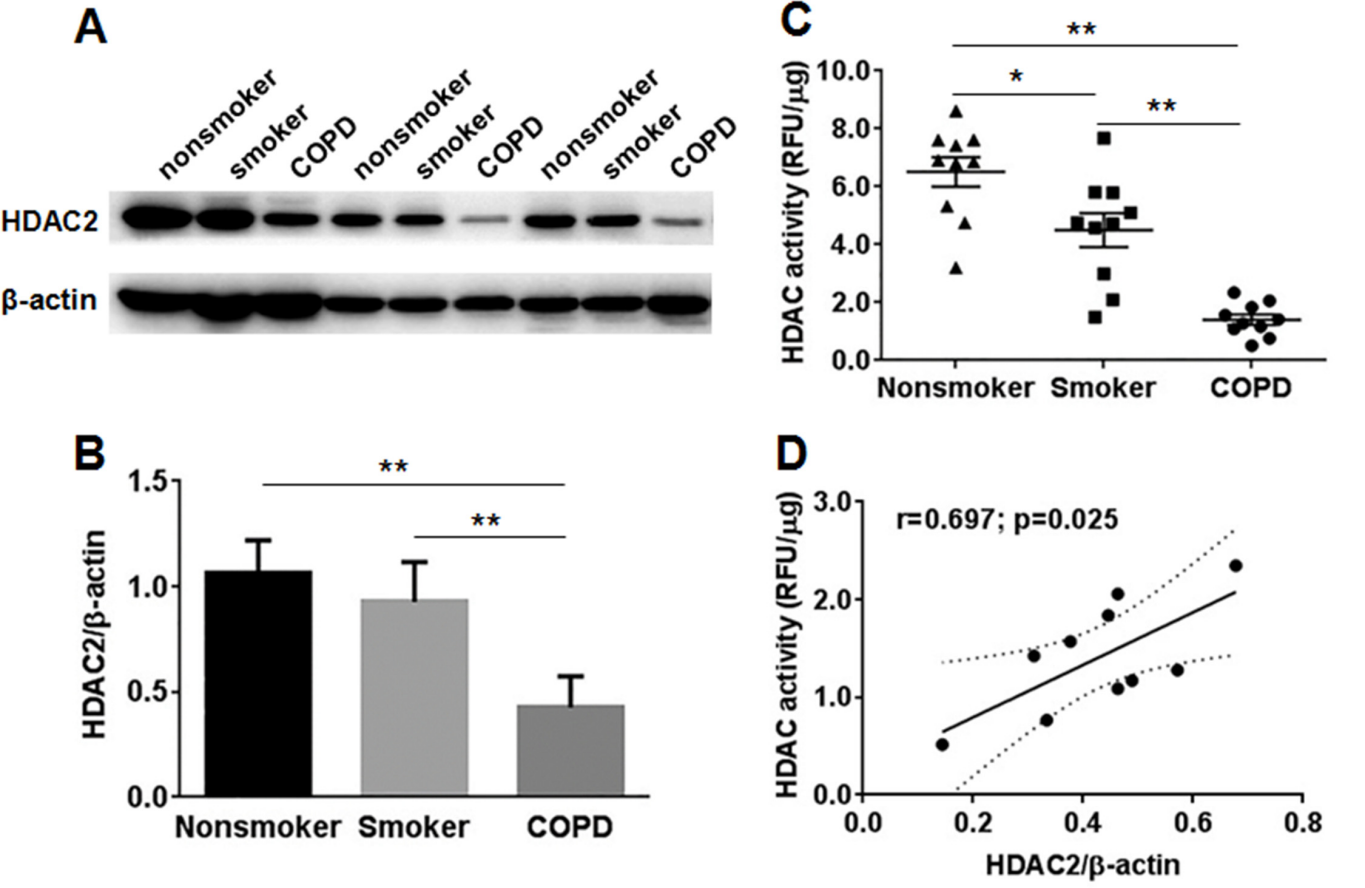
HDAC2 expression and HDAC activity in PBMCs of COPD patients, smokers, and nonsmokers. (A) The HDAC2 protein expression was measured by Western blot in nuclear extracts of PBMCs. β-actin was assessed as a loading control. (B) The relative intensity of HDAC2 was calculated by densitometry. (C) Total HDAC activity in nuclear extracts in PBMCs was measured. (D) Correlation between HDAC2 expression and HDAC activity in nuclear extracts of PBMCs in COPD patients. Data are presented as mean ± SEM. *P < .05, **P < .01. RFU relative fluorescence units.

HDAC2 expression and HDAC activity in PBMCs of COPD patients, smokers and nonsmokers were measured and analyzed. As expected, HDAC2 expression was observed to be reduced markedly in PBMCs of COPD patients compared with smokers (0.43±0.05 vs. 0.93±0.06, P < .0001) and nonsmokers (0.43±0.05 vs. 1.07±0.05, P < .0001) (Fig 1A and 1B). Decreased HDAC activity was also shown in COPD patients compared with smokers (1.41±0.18 RFU/μg vs. 4.51±0.59 RFU/μg, P < .0001) and nonsmokers (1.41±0.18 RFU/μg vs. 40 6.51±0.51 RFU/μg, P < .001) (Fig 1C). Moreover, HDAC2 expression positively correlated with HDAC activity in COPD patients (the correlation coefficient r = 0.697, P = .025) (Fig 1D). In addition, HDAC activity was significantly decreased in PBMCs of smokers compared with nonsmokers (4.51±0.59 vs. 6.51±0.51 RFU/μg, P = .013) (Fig 1C). Thus, our data suggest that HDAC2 expression, as well as HDAC activity, was decreased in PBMCs of COPD patients, which may account for the amplification of inflammatory gene expression.

### Increased NF-κB p65 expression and serum cytokine levels in COPD

**Fig 2:**
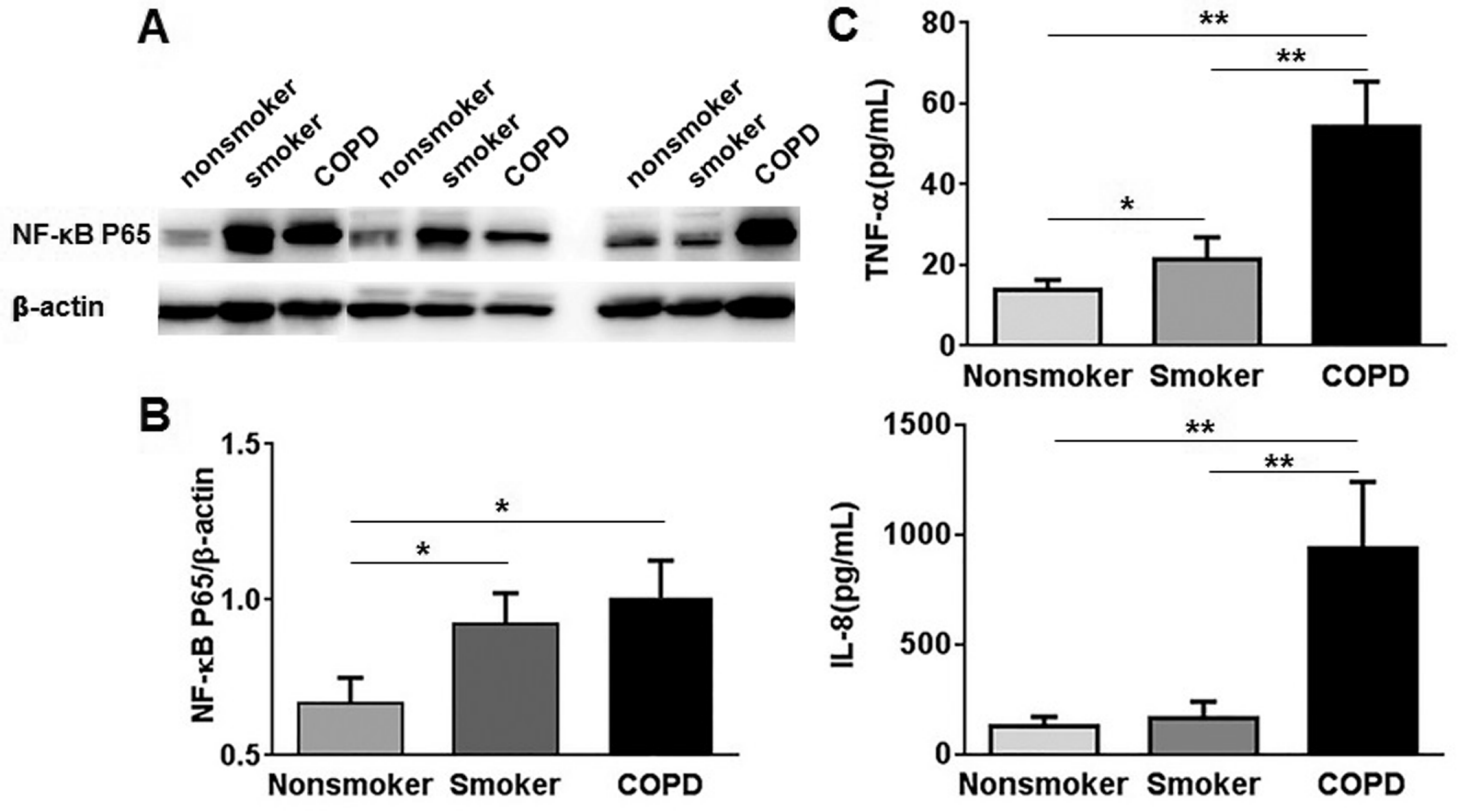
NF-κB p65 expression in PBMCs and serum cytokine levels in nonsmokers, smokers, and COPD patients. (A) The NF-κB p65 protein expression was measured by Western blot in the nuclear extracts of PBMCs. β-actin was assessed as a loading control. (B) The relative intensity of NF-κB p65 was calculated by densitometry. (C) Serum TNF-α level was measured. (D) Serum IL-8 level was measured. Data are presented as mean ±SEM. *P < .05, **P < .01.

Although the mechanism is not fully clarified, previous studies have reported that NF-κB can be activated in the lungs and inflammatory cells, particularly in alveolar macrophages and airway epithelial cells of COPD patients [10, 24]. However, the NF-κB p65 expression in PBMCs of COPD remains unclear. In our study, NF-κB p65 expression was significantly increased in PBMCs of COPD patients compared with nonsmokers (1.01±0.12 vs. 0.63±0.09, P < .05), but not smokers (1.01±0.12 vs. 0.92±0.10, P = .60) (Fig 2A and 2B). Additionally, increased serum TNF-α was shown in COPD patients compared with smokers (54.32±3.52 vs. 21.35±1.73 pg/mL, P<0.0001) and nonsmokers (54.32±3.52 vs. 13.90±0.77 pg/mL, P<0.0001) (Fig 2C). Serum IL-8 was also increased in COPD patients compared with smokers (970.87±74.46 vs. 168.84±23.03 pg/mL, P < .0001) and nonsmokers (970.87±74.46 vs. 132.27±12.58 pg/mL, P < .0001) (Fig 2D). Moreover, NF-κB p65 expression (.92±.10 vs. .63±.09, P < .05) (Fig 2B) and serum TNF-α level (21.35±1.73 pg/mL vs. 13.90±.77 pg/mL, P < .05) (Fig 2C) were also increased in smokers compared with nonsmokers. These results indicate that both stable COPD patients and healthy smokers have increased expression of NF-κB p65 and inflammatory cytokines.

### The severity of COPD was correlated with HDAC2

**Fig 3:**
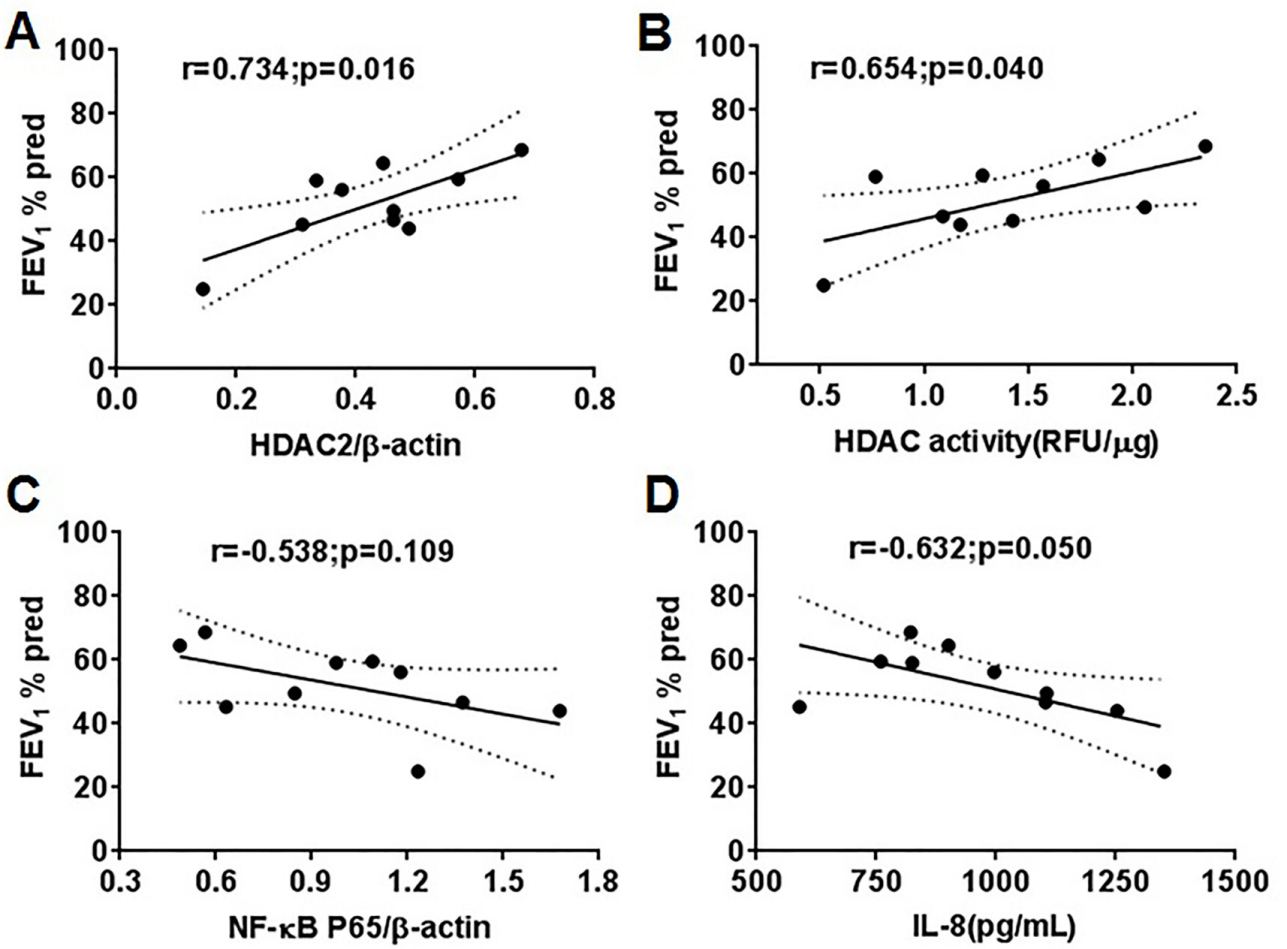
Correlation between FEV_1_%pred with HDAC2 expression, HDAC activity, NF-κB p65 expression and serum cytokine levels in COPD patients. (A) FEV_1_%pred and HDAC2 expression in PMBCs. (B) FEV_1_%pred and HDAC activity in PBMCs. (C) FEV_1_%pred and NF-κB p65 expression in PBMCs. (D) FEV_1_%pred and serum IL-8 level. RFU relative fluorescence units.

Given that HDAC activity may reflect the severity of airway obstruction, the correlation of HDAC2 expression with the severity of airway obstruction in COPD patients was analyzed. The FEV_1_%pred was positively correlated with HDAC2 expression (r = .734, P = .016) (Fig 3A) and HDAC activity (r = .654, P = .040) (Fig 3B). There was a slight but not significant negative correlation between FEV_1_%pred and NF-κB p65 expression (r = -.538, P = .109) (Fig 3C). FEV_1_%pred negatively correlated with serum IL-8 level (r = -.632, P = .050) (Fig 3D). Therefore, HDAC2 expression and HDAC activity in PBMCs could predict the severity of COPD.

## Discussion

The role of HDAC has been previously studied in vitro using specimens from COPD patients [6, 7, 11, 20, 25]. There was a reduction of HDAC activity in alveolar macrophages, bronchial biopsies, bronchoalveolar lavage (BAL) macrophages and PBMCs of COPD [6, 11, 20]. In this present study, we have observed that the HDAC2 protein expression was attenuated in PBMCs of COPD patients. This original finding has not previously been reported. Our data showed that both HDAC2 expression and HDAC activity were inhibited in PBMCs of COPD patients. There is good evidence from clinical studies demonstrating that the change of HDAC2 expression and HDAC activity may contribute to the increase of inflammatory gene expression and corticosteroid insensitivity in COPD [26, 27]. A novel therapeutic strategy may be considered based on the restoration of HDAC2 to reverse corticosteroid resistance in the treatment of COPD.

Chronic inflammation in the airways and lung parenchyma is an important characteristic feature of COPD. The amplification of inflammatory response of COPD was associated with the reduction of HDAC [6, 8–11], which may be secondary to increased oxidants derived from cigarette smoke [9, 14, 25, 28]. In fact, NF-κB also plays a significant role in regulating the expression of inflammatory genes in COPD [18, 22]. NF-κB can be activated by oxidative stress in the lungs and inflammatory cells, and may be affected by HDACs [14, 17, 18, 29, 30]. In the present study, we found that COPD patients and smokers had increased NF-κB p65 expression in PBMCs. That means NF-κB p65 protein was activated in both COPD patients and smokers, which might be activated by cigarette smoke and decreased HDACs [14, 17, 22, 29]. Moreover, the multiple cytokines are also involved in the recruitment and activation of inflammatory cells and contribute to COPD. In our study, beyond the activation of NF-κB p65 protein expression, both serum TNF-α and IL-8 levels were increased in COPD patients. Based on our data, we postulate that the reduction of HDAC2 expression as well as HDAC activity not only directly enhances the expression of inflammatory genes, but may account for the activation of NF-κB and cause the expression of inflammatory mediators in COPD patients, which may be a potential mechanism for the pathophysiology of COPD. Thus, further studies are required to explore the interaction between HDAC2 and NF-κB in COPD patients.

Although Ito and colleagues [6] found that the decreased HDAC activity in alveolar macrophages and lung tissue biopsies was positively correlated with FEV_1_/FVC in COPD patients, no previous studies reported the relationship between HDAC2 expression and lung function in COPD patients. Our data showed that FEV_1_%pred positively correlated with HDAC2 expression and HDAC activity. This result suggests the clinical stage of COPD patients was associated with both HDAC2 expression and HDAC activity in PBMCs, which may be used as a novel biomarker to evaluate the severity of COPD.

Cigarette smoking is the most significant risk factor of COPD. Interestingly, only a minority of smokers (10–15%) develop COPD, suggesting that there may be some characteristics of smokers who develop COPD differentiated from who do not develop COPD [31]. Initials studies have been performed to compare HDAC expression and activity in smokers with and without COPD, but the results of these studies were controversial [5, 9, 20, 32, 33]. To date, no previous studies focused on HDAC2 expression, particularly in PBMCs of smokers with and without COPD. In this study, for the first time, we clearly show that both HDAC2 expression and HDAC activity were decreased in smokers with COPD compared with smokers without COPD. Given that smokers without COPD also had decreased HDAC activity compared with nonsmokers, the reduction of HDAC2 expression might be the biochemical characteristic to identify smokers with COPD from smokers without COPD. Therefore, our study offered a reasonable explanation that why only a few smokers develop COPD.

In addition, relatively little is known about the HDAC and inflammatory response in smokers differentiated from nonsmokers. Previous studies have shown that the HDAC activity was decreased in bronchial biopsies, alveolar and sputum macrophages of smokers compared with nonsmokers [6, 33]. In our study, we found HDAC activity, but not HDAC2 expression was decreased in PBMCs-based assays of smokers. Smokers also had increased NF-κB p65 expression and serum TNF-α compared with nonsmokers. Therefore, our data indicated that cigarette smoking exposure, regardless of COPD, may lead to reduced HDAC activity and increased inflammatory response.

Several limitations of this study should be mentioned. Firstly, the numbers of COPD patients, smokers and nonsmokers were relatively small. Future studies with larger sample sizes are warranted. Secondly, in this study, we didn’t compare current smokers with COPD and ex-smokers with COPD because only two ex-smokers with COPD were included.

## Conclusion

This present study provides novel data on decreased histone deacetylase 2 (HDAC2) in peripheral blood monocytes (PBMCs) of COPD. HDAC2 expression and HDAC activity were decreased in PBMCs of COPD patients, which were both correlated with the severity of the disease. The reduction of HDAC2 expression not only directly enhances the expression of inflammatory genes, but may account for the activation of NF-κB p65 expression and mediate the inflammatory response in COPD. Decreased HDAC2 may serve as a potential biomarker of COPD and predict the decline of lung function.

## Supporting Information

S1 File Correlation between HDAC activity, NF-κB p65 expression and serum cytokine levels in COPD patients and smokers. (A) HDAC activity and NF-κB p65 expression in PBMCs of COPD patients. (B) NF-κB p65 expression in PBMCs and serum IL-8 level of COPD patients. (C) HDAC activity and NF-κB p65 expression in PBMCs of smokers. (D) HDAC activity in PBMCs and serum IL-8 level in smokers. RFU relative fluorescence units.
(TIF)
