# Supplementary figures and images for: Decreased Histone Deacetylase 2 (HDAC2) in Peripheral Blood Monocytes (PBMCs) of COPD Patients
Source: PLoS One. 2016 Jan 25;11(1):e0147380. doi: 10.1371/journal.pone.0147380 (PMC4726592; doi:10.1371/journal.pone.0147380)

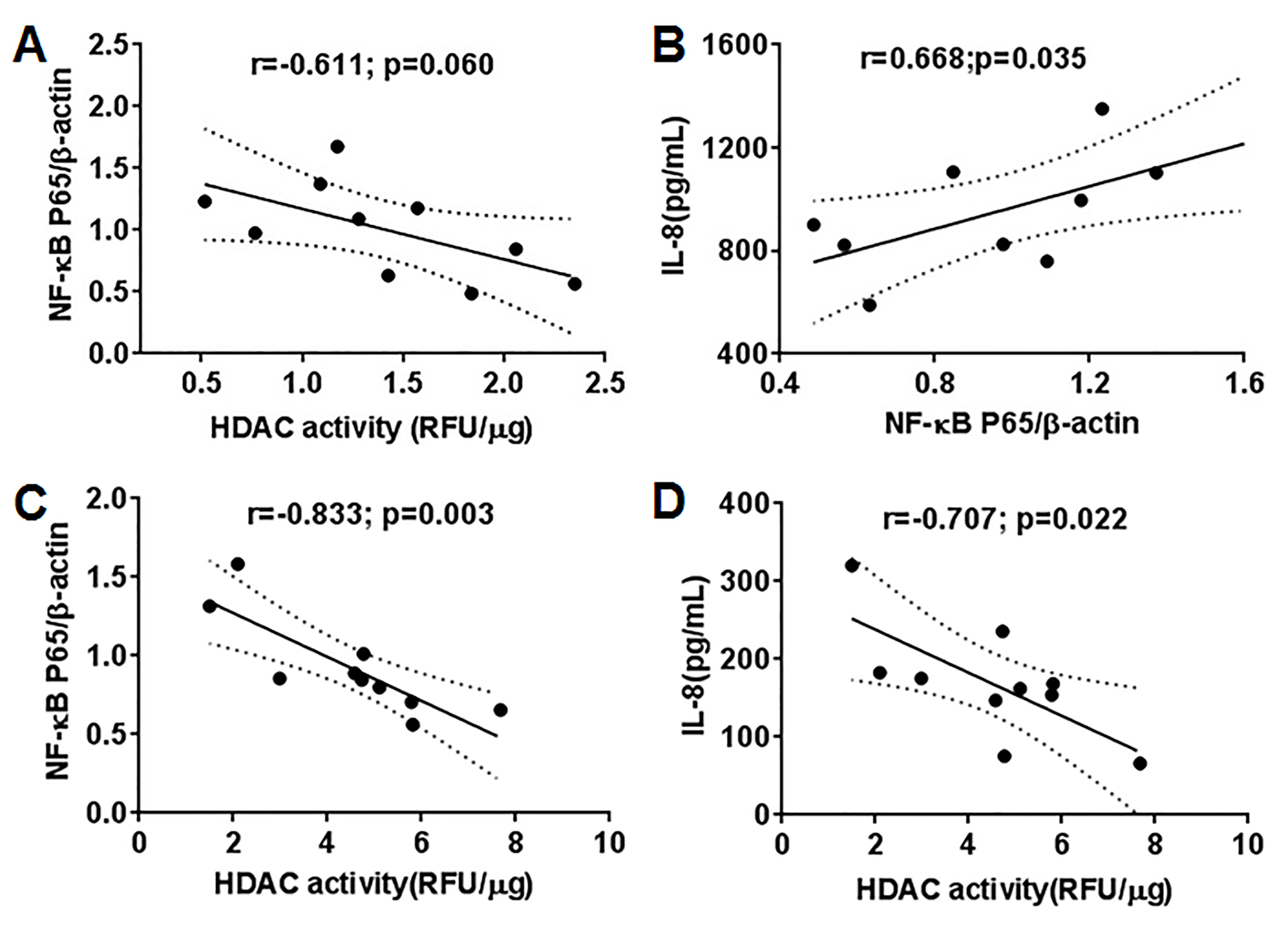

Supplement: S1 File — (A) HDAC activity and NF-κB p65 expression in PBMCs of COPD patients. (B) NF-κB p65 expression in PBMCs and serum IL-8 level of COPD patients. (C) HDAC activity and NF-κB p65 expression in PBMCs of smokers. (D) HDAC activity in PBMCs and serum IL-8 level in smokers. RFU relative fluorescence units. (TIF) [file pone.0147380.s001.tif]
